# Trends in the performance of quality indicators for diabetes care in the community and in diabetes-related health status: an Israeli ecological study

**DOI:** 10.1186/s13584-018-0206-3

**Published:** 2018-01-17

**Authors:** Ronit Calderon-Margalit, Michal Cohen-Dadi, Dana Opas, Dena H. Jaffe, Jacob Levine, Arie Ben-Yehuda, Ora Paltiel, Orly Manor

**Affiliations:** 1 Hadassah-Hebrew University Braun School of Public Health, POB 12272, 9112102 Jerusalem, Israel; 2 Hadassah Medical Organization, Jerusalem, Israel

## Abstract

**Background:**

Israel is one of the few countries that have a national program for quality assessment of community healthcare. We aimed to evaluate whether improved performance in diabetes care was associated with improved health of diabetic patients on a national level.

**Methods:**

We conducted a nationwide ecological study estimating improvements in diabetes-related quality indicators and health outcomes. We estimated both correlations between composite measures of diabetes-related quality indicators and selected outcomes, and assessed through a joinpoint analysis whether trends in selected outcomes changed 4 years after the inception of the national program.

**Results:**

Between 2002 and 2010, the prevalence of diabetes in Israeli adults increased from 4.8% to 7.4%. During these years, an improvement was noticed in most quality indicators (from 53% to 75% for the composite score). Declines were noted in rates of blindness, diabetes-related end-stage kidney disease, lower limbs amputations and diabetes-related mortality. Significant accelerations in decline were noted for amputations in men and diabetes-related mortality in both Arab men and women 4 years after the inception of the national program.

**Conclusion:**

This study suggests that Israel’s national program for quality indicators in diabetes care in the community has probably had a significant impact on the health status of the whole population and may have contributed to narrowing gaps in life expectancy between Israeli Jews and Arabs. Future studies based on individual-level data are needed to confirm these results.

**Electronic supplementary material:**

The online version of this article (10.1186/s13584-018-0206-3) contains supplementary material, which is available to authorized users.

## Background

The measurement of the quality of community healthcare, as a discipline, has evolved during the past two decades, with Israel as one of the few countries to maintain a national program for quality assessment of community healthcare. Since its inception in 2002 and adoption as a national program in 2004, the Israel Quality Indicators in Community Healthcare (QICH) program has monitored community-based healthcare using electronic health records for the entire Israeli population [1]. The mission of QICH is to provide information on the quality of community healthcare in Israel to both policy makers and the public, to promote healthcare monitoring and guideline-based care, and to improve health. The program allows the continuous and dynamic inspection of selected services in the fields of prevention, diagnosis and treatment supplied by the four health maintenance organizations (HMOs).

In Israel, all permanent residents are medically insured under the National Health Insurance Law [2] and are members of one of the four HMOs that supply health services in the community that are included in a nationally determined basket of services. All HMOs support and cooperate with the program, including in the development, assessment and publication of quality indicators. There are no financial incentives or performance-based payments, either to the HMOs or to the physicians [1, 3]. One of the unique features of QICH is that it encompasses the entire Israeli civilian population.

To date, more than 60 quality indicators have been developed, methodically reviewed, and implemented. The major criteria by which these indicators are been evaluated include their importance, validity, and applicability. All indicators are either process measures or intermediate outcome measures.

Quality indicators for diabetes have been the flagship of the QICH program since its establishment. As improved health outcomes is the ultimate goal of health care [3, 4], we aimed to evaluate whether improved conduct of healthcare in diabetes was associated with improved health of diabetic patients on a national level.

## Methods

We conducted an ecological study at the national level. Trends in quality of care between 2002 and 2010 were estimated according to changes in quality indicators developed for diabetes in the adult population with diabetes, who mostly consist of type 2 diabetes. Trends in health outcomes for diabetic patients included outcomes related to target organs (namely, kidneys, lower limbs, and eyes), hospitalizations directly related to diabetes, and mortality from diabetes, irrespective to type of diabetes.

### Quality indicators in diabetes mellitus

Table 1 presents the quality indicators in diabetes used in 2002-2010. These include both process and intermediate outcome measures. The diabetes prevalence measure was based on the prescription of medications for diabetes (either oral hypoglycemics or insulin) and served to determine the denominator population of all diabetes-related process indicators. This measure was changed in 2011 to include laboratory tests; hence, the current data is based on data for 2002-2010. For intermediate outcomes (glycemic control, lipid control, and blood pressure control) denominators were taken from the numerators of the relevant process measures.

**Table 1:** Quality indicators for diabetes mellitus in 2002–2010

Process measures	
Glycemic control documentation	Percentage of diabetics (all ages) with glycemic control (HbA1c) documentation during the measurement year
Cholesterol documentation	Percentage of diabetics (all ages) with cholesterol documentation during the measurement year
Blood pressure documentation	Percentage of adult diabetics aged 18+ years with blood pressure documentation during the measurement year
Eye care documentation	Percentage of diabetics (all ages) with documentation of an eye examination during the measurement year
Kidney function documentation	Percentage of diabetics (all ages) with microalbuminurea documentation during the measurement year
Winter flu shot	Percentage of diabetics aged 5+ years who received the winter flu shot during the winter months of the measurement year (plus 2 months of subsequent year)
BMI documentation	Percentage of adult diabetics aged 18+ years with BMI documentation
Intermediate outcome	
Glycemic control - appropriate control	Percentage of diabetics (all ages) with appropriate glycemic control (HbA1c ≤ 7%) during the measurement year
Inappropriate glycemic control	Percentage of diabetics (all ages) with inappropriate glycemic control (HbA1c > 9%)
Cholesterol – appropriate control	Percentage of diabetics (all ages) with appropriate cholesterol control (≤100 mg/dL) during the measurement year
Blood pressure control	Percentage of adult diabetics aged 18+ years with appropriate blood pressure control (≤130/80 mmHg) during the measurement year (changed to ≤ 140/90 in 2010)

### Data on outcomes

Data were collected for the years 2000 to 2010 or the earliest and latest available within the study period. Sources of information included the Ministry of Health’s Department of Computerized Information for data on hospitalizations and lower limb amputations (1999–2009); The Israeli Center for Disease Control dialysis registry for end-stage renal disease incidence (ESRD, 2002–2010); The Ministry of Welfare for information on blindness (1999–2011); and the Israeli Central Bureau of Statistics for age-standardized rates of diabetes-related mortality (1998–2011) [5]. All but the latter data were retrieved by personal communications.

### Statistical analysis

#### Compliance with individual indicators

**Fig. 1:**
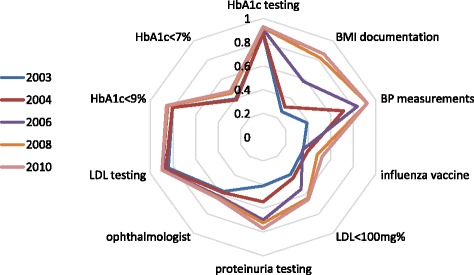
Quality of diabetes care in the community, according to 10 QICH indicators in selected years

The visual representation of composite scores was generated using radar charts in Microsoft Excel. These charts display a graphic representation of multiple performance measures, each measure on a separate axis and all measures per year connected to form a closed area, and provide first-hand monitoring of indicator trending by comparing performance measures overtime (Fig. 1). Each indicator for each year represented the average for the population aged 18 to 74 years. Five data points (2003, 2004, 2006, 2008, and 2010) were chosen to illustrate changes in the rates of adherence for ten QICH diabetes quality indicators. Similar charts were generated for male- and female-specific data (not shown).

#### Composite scores

Composite scores were constructed to give an annual summary for the performance in diabetes-relate quality measure, such that a comparison between years would be more feasible than looking at each indicator separately. Scores were calculated as the area of the radar charts and as the simple average of the specific quality indicators. We calculated composite scores for the ten indicators as well as separate scores for the process and intermediate-outcome measures.

We correlated composite scores with diabetes-related outcomes, including lower extremity amputations and mortality. The composite scores, for the overall and sex-specific populations were based on 2003 to 2010 data. A 2006 starting point for the diabetes-related outcomes was chosen to account for lags in diabetes-related changes as a result of adherence to the QICH diabetes quality indicator set. Data calculated for amputations and mortality represented years 2006 to 2012. Pearson correlation coefficients were calculated in SPSS version 20. Two-sided *P* values are reported for all correlations.

#### Joinpoint analysis

Linear regression models were used to evaluate trends in all outcomes using SAS software version 9.3.We assumed linear trends before and after an index date, and tested whether the trends, as estimated by the coefficients of the linear regression were significantly different before and after an index date, (joinpoint analyses [6]). We chose 2006 as the index date to allow enough latency for improvements in diabetes management promoted by the Program to affect target organ damage and to have enough time points to construct both before and after regressions. We hypothesized that if indeed improvement in treatment for diabetes would lead to a change in trend in morbidity or mortality secondary to diabetes, a latent period of 4 years since the inception of the program would allow sufficient lag time for improvement as well as follow-up time to assess outcomes.

## Results

Between 2002 and 2010 the population of Israeli adults aged 18-74 numbered 4.25 million to 4.76 million. During the study period, the numbers of patients with diabetes increased from 205,725 to 352,747, yielding an increase in prevalence from 4.8% to 7.4%. In 2002, 81% of diabetic patients were tested annually for hemoglobin A1c (HbA1c) and low-density lipoprotein (LDL) cholesterol. By 2010 these rates increased to 93% and 92%, respectively. Annual ophthalmologic examinations increased gradually over time, although not substantially (57% in 2002 and 63% in 2010). During the study period urine testing for proteinuria increased from 35% to 74%. Rates of controlled diabetes among patients 18–74 years old (HbA1c ≤ 7%) increased from 36.9% to 47.5% between 2002 and 2010. Rates of uncontrolled diabetes (HbA1c > 9%) decreased from 22.9% in 2002 to 14.1% in 2010. There was an inverse association between rates of uncontrolled diabetes and age with around 21-30% in those aged 18–54 years and around 10% among those aged 65+ years in 2010. Uncontrolled diabetes rates decreased in almost all age groups. The proportion of diabetes patients with LDL ≤ 100 mg/dl increased from 37.5% in 2002 to 65.0% in 2010.The improvement in process and intermediate outcome measures during the study years is illustrated in Fig. 1, where each closed line represents the levels of all quality indicators in a specific calendar year, and an increase in the area under the curve represents an overall improvement in performance. The composite simple average score increased from 52.8% in 2003 to 75.3% in 2010.

### Outcomes

#### Hospitalizations due to diabetes

The age-adjusted rates of diabetes-related hospitalizations per 100,000 men aged 45 years and older increased from 221 in 1999 to 232 in 2009. In women, these rates decreased from 167 to 149. No significant change in trend was observed in a joinpoint analysis.

#### Dialysis in the general population

During the 2000s, despite a 30% increase in absolute numbers, no changes were noted in rates of ESRD of any cause in the general population (incidence rates per 100,000: 22.7-24.3 in men and 11.2-12.4 in women; see Additional file 1). The number of incident cases for whom diabetes was recorded as the first underlying disease increased by 34% from 473 in 2002 to 638 in 2010; however, there was a much larger increase in the number of patients with diabetes, leading to a decline in rates per 1000 patients with diabetes from 2.28 in 2002 to 1.80 in 2010. No significant change in trend was noted with time.

#### Lower limb amputation due to diabetes

**Fig. 2:**
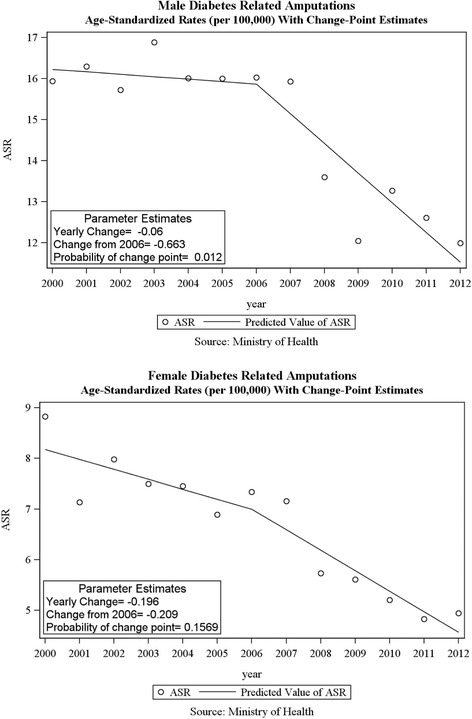
Diabetes-related amputations in males and females – results of the joinpoint analysis

The rate of lower limb amputations due to diabetes has decreased in men from15.9 per 100,000 in 2000 to 12.0 in 2012 (see Additional file 1). The average annual change was - 0.06 per 100,000 until 2006 and - 0.72 per 100,000 per year thereafter (P for change = 0.01). Although among women rates of amputations declined from 8.83 to 4.94 per 100,000 with an acceleration of decline from - 0.20 to - 0.41 per 100,000, this acceleration did not reach statistical significance (*p* = 0.16) [Fig. 2]. A significant correlation was found between composite scores and amputation rates (correlation coefficient: - 0.882, *p* = 0.020).

#### Blindness due to diabetes

The rate of blindness due to diabetes decreased from 1.48 to 0.39 per 1000 diabetic patients (see Additional file 1). The rate of decrease was steady throughout the study period with no significant change after 2006. However, unlike the case of dialysis due to diabetes, numbers of incident cases halved between 1999 and 2000 and 2011 (from around 300 to 150 new cases annually, respectively).

#### Diabetes mortality

**Fig. 3:**
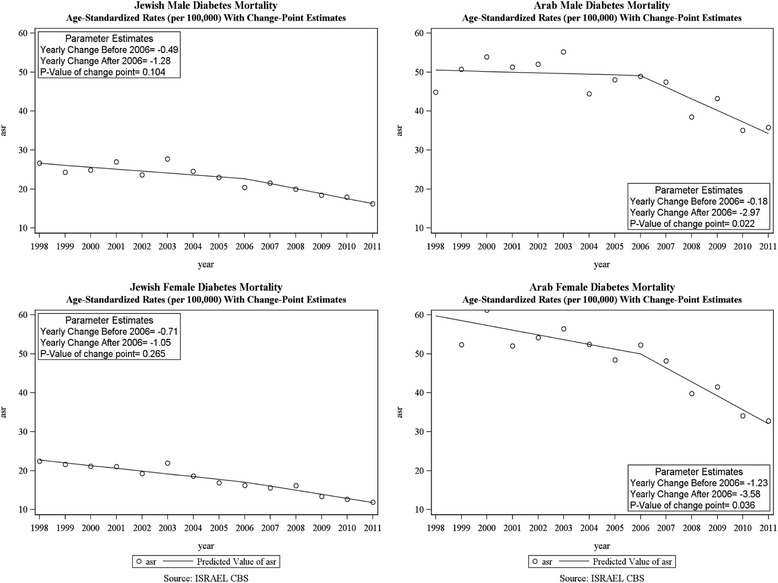
Mortality due to diabetes, in Jewish and Arab males and females, 1998–2011

The age-adjusted death rates due to diabetes per 100,000 decreased in Jewish males from 26.7 in 1998 to 16.2 in 2011 (see Additional file 1). Among Arab males these rates decreased from 44.8 to 35.8, respectively. In Jewish females the respective death rates were 22.4 and 11.9, and in Arab women these rates were 64.6 and 32.8, respectively. In linear regressions with joinpoint analysis, the decrease has significantly accelerated after 2006 in both Arab males and females (from annual change of - 0.18 to - 2.97 per 100,000 in Arab males, *P* = 0.022, and from - 1.23 to - 3.58 per 100,000 in females, *P* = 0.036 [Fig. 3]).Significant correlations were found between composite scores and diabetes-related mortality in the whole population (correlation coefficients: -0.990, *P* < 0.001).

## Discussion

We demonstrated that improvements in diabetes care in the community were associated with improved health. Specifically, these achievements consisted of accelerated decreases in lower limb amputations in men and in mortality due to diabetes in the Arab population.

Our data on a decrease in amputation rates is in agreement with other studies that have shown a decrease in amputations among patients with diabetes in Denmark and Canada in center-based studies [7]. Similarly, in a population-based cohort study in Denmark, a significant decrease in amputations in diabetic patients was noted [8]. A study from England demonstrated a non-significant decrease in rates of diabetes-related amputations between 2004 and 2008, whereas among non-diabetic individuals there was a significant decrease in both numbers and rates [9]. Our study further suggests an acceleration in the decrease of diabetes-related amputations 4 years after the inception of QICH and a negative correlation between a summary measure of diabetes-related quality of care and rates of amputations.

The present study supports and extends a previous report on a decline in diabetes-related blindness in Israel [10]. In the US, the number of diabetic individuals who reported having visual impairment increased between 1997 and 2010; however, the age-adjusted rate has decreased from 24% to 17% between 1997 and 2006 and remained relatively stable since then. There was no change in rates of ophthalmological visits among those with or without visual impairment [11]. Although we did not observe a change in trends of blindness following the establishment of the QICH program, there was a decrease both in absolute numbers and in rates that could not be attributed to changes in ophthalmological practices. Rather, it seems that treatment of dyslipidemia and HbA1c may had had an impact in prevention of diabetes related blindness [12]. Nevertheless, there may be other explanations for that trend, as blindness due to other causes has decreased as well during these years [10].

Our study could not support a decrease in diabetes-related nephropathy. A study from the Netherlands, where the total population was used as the denominator, showed a decrease in incidence rates of ESRD related to type 1 diabetes, whereas there was an increase in ESRD related to type 2 diabetes between 2000 and 2012 [13]. In a study from the US, a decrease was demonstrated in the diabetes-related ESRD between 1990 and 2006. However, similar to our results regarding diabetes-related ESRD [14], the denominator used was of the estimated number of diabetic patients and there was a substantial increase in absolute numbers, both in diabetes-related ESRD and in ESRD of any cause. Similarly, the proportion of diabetes among new cases of ESRD remained stable, suggesting that the decrease in diabetes-related ESRD was merely due to the increase in the number of individuals defined as diabetics and did not necessarily reflect better prognosis for diabetic patients. Nevertheless, a study from 18 European countries and regions, where the general population served as the denominator, demonstrated an increase in diabetes-related renal replacement therapy (RRT) in 2001-2007 and a decrease thereafter (2007–2011) [15].

We observed an annual decrease in mortality from diabetes in both men and women (- 3.77% and - 3.85%, respectively). Smaller declines in mortality rates from diabetes were shown in the US (- 2.8% between 2002 and 2010) [16]. A previous study in one of the Israeli HMOs demonstrated an association between glycemic control and mortality [17].We did observe a significant acceleration in this decrease in the Israeli Arab population. Unfortunately, data on ethnicity is not collected at the national level and therefore, this assessment was not available for the QICH data. Nevertheless, it has been shown that the prevalence of type 2 diabetes is much higher among the Arab population compared with the Jewish population in Israel [18], partly explained by the higher prevalence of obesity among Israeli Arabs population compared with Jews and especially among women [19, 20]. Diabetes mortality is one of the leading contributors to gaps in life expectancy between Israeli Arabs and Jews, with increasing gaps attributed to diabetes mortality in the years 1980–2004 [21]. A more recent analysis demonstrated very low rates of diabetes mortality in the 1980s that increased drastically in the 1990s and 2000s, in both Jews and non-Jews; however, steeper increases were shown among Arab men and women. Among Arab women, diabetes mortality became the major contributor to the Arab-Jewish gap in life expectancy at age 45 [21], although even in this latter study, the beginning of a declining trend was starting to show. The most recent publication by the Israeli Central Bureau of Statistics has demonstrated the smallest Jewish-Arab gap in life expectancy at age 45 among women since 2000 [22]. Previous studies from Israel’s two largest HMOs described the efforts invested in improving the quality of primary healthcare supplied to Arabs and individuals of low socioeconomic status to minimize inequalities [23–25]. Our study suggests that these efforts have yielded improved outcomes.

Our study’s main limitation is its ecological design, which prevents causal inference, since associations shown on the national level may not exist at the individual level. Furthermore, our study cannot specify what interventions or medications were responsible for the improvements in the quality indicators, nor could we gather information by type of diabetes, specific cause of hospitalization, or other clinically important outcomes, such as cardiovascular diseases in patients with diabetes. The short time frame of the study did not allow us to evaluate inflection points other than 2006. Future studies are needed to support our findings in a population-based cohort study. Nevertheless, the strength of this study is in its national scope.

## Conclusion

We showed that programs for improvement of quality care, such as the QICH, have a larger effect than data collection since they initiate and support ongoing efforts of healthcare providers to improve the healthcare supplied. This, in turn, improves the health status of their clients, and in this case, of the whole country. Future studies should extend the time frame of this study and include individual-based cohort studies.

## Additional file

Additional file 1: Supplementary Table: Age-standardized incidence rates of selected diabetes-related outcomes in the Israeli population, by year. (DOCX 27 kb)

## Abbreviations

(ESRD): End-stage renal disease incidence
(HbA1c): Hemoglobin A1c
(HMO): Health maintenance organizations
(LDL): Low-density lipoprotein
(QICH): Israel Quality Indicators in Community Healthcare program
