# Supplementary material for: Trends in the performance of quality indicators for diabetes care in the community and in diabetes-related health status: an Israeli ecological study
Source: Isr J Health Policy Res. 2018 Jan 17;7:10. doi: 10.1186/s13584-018-0206-3 (PMC5773014; doi:10.1186/s13584-018-0206-3)
Supplement: Additional file 1: — Supplementary Table: Age-standardized incidence rates of selected diabetes-related outcomes in the Israeli population, by year. (DOCX 27 kb) [file 13584_2018_206_MOESM1_ESM.docx]

Table: Age-standardized incidence rates of selected diabetes-related outcomes in the Israeli population, by year

|  | 2000 | 2001 | 2002 | 2003 | 2004 | 2005 | 2006 | 2007 | 2008 | 2009 | 2010 | 2011 | 2012 |
| --- | --- | --- | --- | --- | --- | --- | --- | --- | --- | --- | --- | --- | --- |
| **Lower-limb amputation**  Per 100,000 | | |  |  |  |  |  |  |  |  |  |  |  |
| males | 15.94 | 16.30 | 15.73 | 16.88 | 16.01 | 16.00 | 16.03 | 15.93 | 13.60 | 12.05 | 13.28 | 12.61 | 12.00 |
| females | 8.83 | 7.13 | 7.98 | 7.49 | 7.45 | 6.89 | 7.34 | 7.15 | 5.73 | 5.61 | 5.20 | 4.83 | 4.95 |
| **ESRD**  Per 100,000 |  |  |  |  |  |  |  |  |  |  |  |  |  |
| males | 22.70 | 22.80 | 23.18 | 23.73 | 22.96 | 23.13 | 23.38 | 24.35 | 22.89 | 23.34 | 22.79 |  |  |
| females | 12.00 | 12.53 | 11.99 | 12.29 | 12.91 | 11.89 | 12.44 | 11.83 | 12.00 | 12.43 | 11.22 |  |  |
|  |  |  |  |  |  |  |  |  |  |  |  |  |  |
| **Blindness**  Per 1,000 diabetic patients | NA | NA | 1.48 | 1.27 | 1.17 | 1.23 | 0.87 | 0.79 | 0.65 | 0.51 | 0.38 | 0.38 |  |
| **Mortality** |  |  |  |  |  |  |  |  |  |  |  |  |  |
| Males |  |  |  |  |  |  |  |  |  |  |  |  |  |
| Jews | 24.8 | 27.0 | 23.6 | 27.7 | 24.5 | 22.9 | 20.4 | 21.5 | 20.0 | 18.4 | 17.9 | 16.2 |  |
| Arabs | 53.9 | 51.3 | 52.0 | 55.2 | 44.4 | 48.0 | 48.9 | 47.5 | 38.4 | 43.2 | 35.0 | 35.8 |  |
| Females |  |  |  |  |  |  |  |  |  |  |  |  |  |
| Jews | 21.1 | 21.1 | 19.2 | 21.9 | 18.6 | 16.9 | 16.3 | 15.5 | 16.2 | 13.4 | 12.6 | 11.9 |  |
| Arabs | 61.2 | 52.0 | 54.1 | 56.4 | 52.4 | 48.4 | 52.2 | 48.2 | 39.8 | 41.5 | 34.1 | 32.8 |  |
